# Outcomes of Percutaneous Tracheostomy for Patients With SARS-CoV-2 Respiratory Failure

**DOI:** 10.1097/LBR.0000000000000854

**Published:** 2022-04-05

**Authors:** Jason Arnold, Catherine A. Gao, Elizabeth Malsin, Kristy Todd, Angela Christine Argento, Michael Cuttica, John M. Coleman, Richard G. Wunderink, Sean B. Smith

**Affiliations:** * Department of Internal Medicine; † Division of Pulmonary and Critical Care Medicine, Northwestern University Feinberg School of Medicine, Chicago, IL

**Keywords:** COVID-19, SARS-CoV-2, tracheostomy, percutaneous tracheostomy, interventional pulmonology

## Abstract

Severe acute respiratory syndrome coronavirus 2 (SARS-CoV-2) can cause severe respiratory failure leading to prolonged mechanical ventilation. Data are just emerging about the practice and outcomes of tracheostomy in these patients. We reviewed our experience with tracheostomies for SARS-CoV-2.

**Methods:**

We retrospectively reviewed the demographics, comorbidities, timing of mechanical ventilation, tracheostomy, and intensive care unit and hospital lengths of stay in SARS-CoV-2 patients who received tracheostomies performed by the interventional pulmonary team. A tertiary care, teaching hospital in Chicago, Illinois. From March 2020 to April 2021, our center had 473 patients intubated for SARS-CoV-2, and 72 (15%) had percutaneous bedside tracheostomy performed by the interventional pulmonary team.

**Results:**

Median time from intubation to tracheostomy was 20 (interquartile range: 16 to 25) days. Demographics and comorbidities were similar between early and late tracheostomy, but early tracheostomy was associated with shorter intensive care unit lengths of stay and a shorter total duration of ventilation. To date, 39 (54%) patients have been decannulated, 17 (24%) before hospital discharge; median time to decannulation was 22 (IQR: 18 to 36) days. Patients that were decannulated were younger (56 vs. 69 y). The rate of decannulation for survivors was 82%. No providers developed symptoms or tested positive for SARS-CoV-2.

**Conclusion:**

Tracheostomy enhances care for patients with prolonged respiratory failure from SARS-CoV-2 since early tracheostomy is associated with shorter duration of critical care, and decannulation rates are high for survivors. It furthermore appears safe for both patients and operators.

Severe acute respiratory syndrome coronavirus 2 (SARS-CoV-2) infection may cause acute, severe respiratory failure that can lead to prolonged mechanical ventilation. At the onset of the pandemic there were questions regarding the safety and value of tracheostomy for SARS-CoV-2 patients with prolonged respiratory failure. Tracheostomy has since been described and safely performed in these patients, and several societal guidelines support performing tracheostomy with proper personal protective equipment and precautions.^1^–^6^

Data are emerging about the practice and outcomes of tracheostomy for prolonged respiratory failure from SARS-CoV-2. Rates of decannulation and survival have varied in the literature. In the largest cohorts from Spain and England, median times to tracheostomy ranged from 12 to 16 days.^7^,^8^ The Spanish multicenter cohort found an overall mortality rate of 23%, and their decannulation rate for survivors weaned from ventilation was 81%.^7^ Another study from England reported 85% survival at 30 days and a 99% overall decannulation rate for survivors.^9^ Several smaller studies from earlier in the pandemic reported lower decannulation rates, ranging from 8% to 13%.^1^,^3^,^4^ However, one of the largest systematic review and meta-analysis comprising over 3000 patients found an average decannulation rate of 34.9%.^7^ One potential explanation for differences is lack of availability and willingness of long-term acute care hospital (LTACH) facilities to accept SARS-CoV-2 infected patients, wanting to wait for viral clearance before accepting patients for transfer. Thus, patients had longer overall hospital stays and a longer opportunity to be decannulated during the same hospital admission.

We sought to review our practice at a large, US tertiary care, urban teaching hospital that has had a high volume of patients with SARS-CoV-2 respiratory failure. Our goals were to determine mortality and decannulation rates as well to assess patient characteristics associated with successful outcomes.

## METHODS

We reviewed patients with SARS-CoV-2 who had percutaneous bedside tracheostomy performed by the Interventional Pulmonary team for prolonged respiratory failure in our single-center, tertiary care, urban teaching hospital from March 2020 to April 2021. Percutaneous tracheostomies were performed at the bedside by providers wearing powered air purifying respirators, gowns, and gloves. The procedures followed our standard practices for percutaneous tracheostomy, with additional steps taken to minimize aerosolization including packing the oropharynx with gauze to minimize aerosolization when the cuff on the endotracheal tube was deflated. Patient demographics and comorbidities, the timing of mechanical ventilation and tracheostomy, as well as intensive care unit (ICU) and hospital lengths of stay (LOS) were cataloged. Primary outcomes included overall mortality and decannulation rates, whereas the secondary outcome was time to weaning from mechanical ventilation. The timing of tracheostomy was at the discretion of the ICU attending and interventional pulmonologist who performed the procedures. Tracheostomy was considered early when performed within 14 days of initiation of mechanical ventilation. Like other centers, we experienced 2 waves of admissions: we considered the first wave those admitted from March to July 2020; whereas the second wave included those admitted after August 1, 2020. This study was reviewed and approved by the Institutional Review Board, STU00212283.

Statistical analyses were performed with STATA 11.2 (College Station, TX). Not all continuous data were normally distributed, and so median values with interquartile ranges (IQR) were calculated. Nonparametric analyses included Wilcoxon rank sum and the Spearman correlation testing. Kruskal-Wallis testing was used to compare data across multiple categories. Regression modeling was used to identify variables associated with outcomes. Statistical significance was established as *P*<0.05.

## RESULTS

From March 2020 to April 2021, 473 patients were intubated for SARS-CoV-2 respiratory failure, and percutaneous bedside tracheostomy by the interventional pulmonary group was performed in 72 (15%) patients. Median age was 66 (IQR: 58 to 71) years, and 29% were female. Median body mass index (BMI) was 26 (24 to 31), and the median number of comorbidities was 2 (IQR: 1 to 3). Additional patient characteristics and demographics are listed in Table 1. The most common comorbidities (Table 2) were hypertension (61%), diabetes (50%), and obesity (35%). Thirty-eight patients (58%) were treated with steroids, and 30 (42%) with remdesivir, either under the emergency use authorization or following US Food and Drug Administration (FDA) approval. An additional 5 (7%) patients participated in a double blind remdesivir versus placebo trial. Two patients underwent extracorporeal membrane oxygenation (ECMO) and had tracheostomies placed by the interventional pulmonary group. Other patients who underwent ECMO had tracheostomies placed by the thoracic surgery group and were excluded from our analysis.

**TABLE 1:** Baseline Characteristics of Patients With SARS-CoV-2 Who Had Tracheostomy

	Total/Median	IQR/Percent
Patients	72	
Age	66	58-71
Female	21	29
Race/ethnicity		
African American	29	40
Asian	3	4
Caucasian	19	26
Hispanic	18	25
Other	3	4
Comorbidities	2	1-3
BMI	26.0	24.0-31.0
Smoking status		
Active smoker	6	8
Former smoker	23	32
Time from intubation to tracheostomy	20	16-25
Oxygenation on day of tracheostomy		
PEEP	8	5-10
FiO_2_	40%	40-50%
Treatments		
Remdesivir	30	42
Steroids	38	53
ECMO utilization	2	3
Type of tracheostomy tube		
Shiley 6	25	35
Shiley 6 proximal XLT	2	3
Shiley 6 distal XLT	42	58
Shiley 8	2	3
Bivona 6 TTS	1	1

BMI indicates body mass index; ECMO, extracorporeal membrane oxygenation; IQR, interquartile range; PEEP, positive end expiratory pressure; SARS-CoV-2, severe acute respiratory syndrome coronavirus 2; TTS, tight to shaft; XLT, extended length tracheostomy.

**TABLE 2:** Comorbidities of Patients With SARS-CoV-2 Who Had Tracheostomy

Comorbidities	N (%)
Hypertension	44 (61)
Diabetes mellitus	36 (50)
Obesity	24 (35)
Coronary artery disease	14 (19)
Chronic kidney disease	13 (18)
Solid organ malignancy	10 (14)
Heart failure	10 (14)
Atrial fibrillation	9 (13)
Obstructive sleep apnea	8 (11)
Cerebrovascular disease	6 (8)
Chronic lung disease	5 (7)
Solid organ transplantation	5 (7)
End-stage renal disease	5 (7)
Deep vein thrombosis/pulmonary embolism	4 (6)
Cirrhosis	2 (3)
Hematologic malignancy	2 (3)

SARS-CoV-2 indicates severe acute respiratory syndrome coronavirus 2.

Median time from intubation to tracheostomy was 20 (IQR: 16 to 25) days. Median positive end expiratory pressure was 8 (IQR: 5 to 10), and FiO_2_ was 40% (40% to 50%) on the day of tracheostomy. The most common tracheostomy placed was a Shiley 6 distal extended-length tracheostomy (XLT) (n=42, 58%). No procedural complications related to tracheostomy placement occurred. The most common overall hospital complications (Table 3) were pneumonia (83%), and most of the pneumonia cases (45/60) occurred before tracheostomy placement. Venous thromboembolism (60%), acute renal failure requiring dialysis (38%), and pneumothorax (29%) were the other common hospital complications.

**TABLE 3:** Hospital Complications of Patients With SARS-CoV-2 Who Had Tracheostomy

Complications	N (%)
Pneumonia	60 (83)
Pneumonia before tracheostomy	45 (63)
Deep vein thrombosis/pulmonary embolism	43 (60)
Acute renal failure requiring hemodialysis	27 (38)
Pneumothorax	21 (29)
Atrial or ventricular arrhythmia	16 (22)
Bacteremia	8 (11)
Hemorrhage	8 (11)
Acute stroke	8 (11)
Cardiac arrest	7 (10)
Cardiomyopathy	5 (7)
Diabetic ketoacidosis	3 (4)
Rhabdomyolysis	2 (3)
Tracheal stenosis	2 (3)
Cardiac tamponade	1 (1)

SARS-CoV-2 indicates severe acute respiratory syndrome coronavirus 2.

Outcomes (Table 4) include median length of follow-up of 45 (IQR: 16 to 135) days, with 71% of patients having 30-day follow-up data available. Decannulation occurred in 39 patients (82% of survivors and 54% of all patients), with 17 patients (44%) being decannulated before hospital discharge. Median time to decannulation was 22 (IQR: 18 to 36) days. Median ICU LOS was 38 (IQR: 32 to 44) days, and hospital LOS was 42 (IQR: 35 to 56) days. Median duration of mechanical ventilation was 35 (IQR: 31 to 41) days, and median time from tracheostomy to weaning from mechanical ventilation was 19 (IQR: 12 to 20) days.

**TABLE 4:** Outcomes of Patients With SARS-CoV-2 Who Had Tracheostomy

Outcomes	Total/Median	IQR/Percent
Length of follow-up	45	16-135
30 d follow-up available	51	71
Decannulated	39	54
Decannulated by discharge	17	24
Decannulated survivors	36	82
Time to decannulation	22	18-36
Hospital length of stay	42	35-56
Intensive care unit length of stay	38	32-44
Duration of ventilation	35	31-41
Time from tracheostomy to ventilator wean	19	12-20
Weaned from ventilator	42	58
Weaned by discharge	32	44
Mortality	28	39
In hospital mortality	23	32
Disposition		
Long-term acute care facility	30	42
Died in hospital	23	32
Home	11	15
Acute inpatient rehabilitation	9	13

SARS-CoV-2 indicates severe acute respiratory syndrome coronavirus 2.

Overall mortality was 39%, and hospital mortality was 32%. Those patients that died were older in age [69 (IQR: 65 to 71) vs. 65 (55 to 71), *P*=0.04] and had a lower BMI [26 (24 to 28) vs. 28 (24 to 35), *P*=0.04], and higher FiO_2_ at time of tracheostomy [45% (40% to 50%) vs. 40% (40% to 50%), *P*=0.04]. Only presence of a pneumothorax (odds ratio: 2.92, 1.02-8.31, *P*=0.04) was associated with overall mortality.

Demographics, comorbidities, and ventilator settings were similar for those who had tracheotomy before or after 14 days of mechanical ventilation. Early tracheostomy was associated with shorter ICU duration [29 (IQR: 27 to 38) vs. 39 (35 to 46) days, *P*=0.007], and a shorter duration of mechanical ventilation [31 (24 to 34) vs. 36 (32 to 46) days, *P*=0.005]. Both early and late tracheostomy groups had similar tracheostomy-to-wean days (median 19 d). Therefore, earlier tracheostomy was associated with decreased total duration of mechanical ventilation. Neither survival nor decannulation rates differed between early or late tracheostomy (Table 5).

**TABLE 5:** Subgroup Analysis Comparing Early Tracheostomy (Trach Placement Before 14 Days of Intubation) With Late (>14 d of Intubation) Tracheostomy Placement

Outcomes	Early Tracheostomy	Late Tracheostomy	*P*
Day of intubation tracheostomy placed	12 (11-14)	22 (19-28)	<0.0001
Weaned from ventilator by discharge	50%	43%	0.86
Duration of mechanical ventilation	31 (24-34)	36 (32-46)	0.005
Time from tracheostomy to ventilator wean	19 (9-23)	19 (11-20)	0.96
Decannulated	64%	52%	0.59
Hospital length of stay	37 (32-57)	43 (37-54)	0.25
Intensive care unit length of stay	29 (27-38)	39 (35-46)	0.007
Mortality	29%	41%	0.57
In hospital mortality	29%	33%	1.00

Patients who were decannulated were younger [65 (IQR: 55 to 71) vs. 69 (65 to 72) years, *P*=0.01] and had higher BMI [28 (24 to 35) vs. 26 (24 to 29), *P*=0.04]. Seventy-three percent of survivors were weaned from mechanical ventilation by hospital discharge. Demographics, comorbidities, and tracheostomy characteristics were not associated with time to weaning. Late tracheostomy placement (*P*=0.005) and development of in-hospital pneumonia (*P*=0.03) were associated with a longer time on mechanical ventilation.

No providers involved in the placement of tracheostomies developed a SARS-CoV-2 infection during our study period. Our hospital used a testing system based on the development of symptoms.

### Subgroup Analysis by Admission Date

There were 46 patients who had tracheostomies in the first wave and 26 in the second wave (Table 6). Baseline demographics (ie, age, sex, comorbidities) and times from intubation to tracheostomy were similar between the 2 waves. There was significantly higher utilization of steroids (96.2% vs. 28.3%, *P*<0.0001) and remdesivir (80.8% vs. 30.4%, *P*<0.0001) in the second wave. In hospital mortality was higher in the second wave of the pandemic 50% vs 21% in the first wave (*P*=0.02). Rates of renal failure and pneumonia were similar, but there were higher rates of pneumothorax (46.2% vs. 19.6%, *P*=0.02) and venous thromboemboli (76.9% vs. 50.0%, *P*=0.02) in the second wave. Hospital LOS, ICU LOS, duration of mechanical ventilation, and time to weaning from mechanical ventilation were similar between the 2 waves. Decannulation by hospital discharge was higher in the first wave (37.0% vs. 0%, *P*=0.0004).

**TABLE 6:** Subgroup Analysis Comparing Wave 1 and Wave 2 of the Coronavirus Pandemic

Outcomes	Wave 1	Wave 2	*P*
Patients	46	26	—
Remdesivir use	30.4%	80.8%	<0.0001
Steroid administration	28.3%	96.2%	<0.0001
Decannulated by discharge	37.0%	0%	0.0004
Hospital length of stay	41 (35-54)	44 (38-63)	0.22
Intensive care unit length of stay	37 (32-42)	41 (35-47)	0.13
In hospital mortality	21%	50%	0.02

## DISCUSSION

We found that tracheostomy for SARS-CoV-2 patients was a safe and reasonable practice for prolonged respiratory failure. As described in similar studies, we found no incidents of operators contracting SARS-CoV-2 infection during tracheostomy.^2^,^5^,^6^,^8^–^10^

The ideal time from intubation to tracheostomy for SARS-CoV-2 has been debated, as it has been for other critical illnesses, and there remains no consensus recommendation.^11^ There had been initial concerns that tracheostomy should be delayed until after active SARS-CoV-2 viral replication, whereas others proposed early tracheostomy to facilitate weaning and preserve resources during pandemic.^12^ Our practice has been to maintain traditional standards for tracheostomy selection with regard to timing, oxygenation (FiO_2_≤50%, positive end expiratory pressure≤10), and hemodynamic stability, without a particular delay in timing because of a patient’s SARS-CoV-2 status. Although we do not have data regarding sedation dosing before and after tracheostomy, we suspect that tracheostomy facilitates the lightening of sedation and faster weaning from mechanical ventilation SARS-CoV-2 patients as in other causes of prolonged respiratory failure.^7^ We started by placing Shiley 6 tracheostomies as others have, but often found that patients developed significant cuff leaks with this option, and required a switch to Shiley 6 distal XLT tracheostomies. This may have been because of tracheomalacia from prolonged mechanical ventilation. We thus moved to placing Shiley 6 distal XLT tracheostomies as our default device.^13^,^14^

Our median times to tracheostomy (19 d) and times to weaning (17 d) are similar to other reports in the literature.^2^–^4^,^15^,^16^ Mata-Castro et al^16^ found that a longer time from intubation to tracheostomy was related to a longer time from tracheostomy to weaning. Two other studies found that early tracheostomy was associated with shorter overall duration of mechanical ventilation and ICU LOS.^2^,^6^ From our data, we find that tracheostomy placement before 14 days of intubation was associated with shorter ICU stays and shorter durations of mechanical ventilation. This associated decrease in duration of mechanical ventilation was likely driven primarily by the earlier tracheostomy placement, as both early and late tracheostomy groups had similar tracheostomy-to-wean days of median 19 days.

Differences between the initial surge in the spring of 2020 and a second wave later in the fall have been described. Subgroup analyses between our 2 waves found trends towards more pneumothorax and venous thromboemboli in the second wave and undoubtedly more patients received steroids in the second wave because of the RECOVERY trial.^17^ Mortality was also higher in the second wave.

In our analysis, it was found that pneumothorax was associated with an increased risk of death for patients who had received tracheostomy for severe SARS-CoV-2 infection. Similar findings were described in a case-control study of pneumothorax in Spain. This study included all-comers who presented with coronavirus disease (COVID) infection to the emergency department found that pneumothorax in patients with COVID was associated with more severe disease, level of inflammation and increased hospital mortality (odds ratio: 15).^18^

Studies have demonstrated overall relatively good survival rates for patients with SARS-CoV-2 who had tracheostomy, with mortality ranging from 7% to 23%.^1^,^3^,^5^ Many studies have been affected by duration of follow-up and available outcome data, and the largest study from Spain reports one of the higher mortality rates of 23%.^7^ Complete 30-day follow-up data were available for 70% of our patients, and this subgroup had a 38% 30-day mortality rate. This elevation in mortality found in our study may be attributed to the high number of external hospital transfers (n=12, 17%), who were sicker than our in-house population, as they were transferred for escalation of care and consideration of ECMO. Most patients cannulated for ECMO had tracheostomies performed by the thoracic surgery department and were excluded from our analyses. Those that were rejected for ECMO and required tracheostomy were often placed by the interventional pulmonary team and included in the study. Survival amongst tracheostomy patients has been described as higher than those who did not receive tracheostomy,^9^ although selection bias is to be considered for which patients are stable enough to have tracheostomy.

Long-term outcomes of lung function are unknown for patients who survive SARS-CoV-2 respiratory failure. Short-term decannulation rates for non-SARS-CoV-2 ARDS are not well published, but already multiple studies, including ours, have demonstrated high rates of decannulation within only a few months after ARDS from SARS-CoV-2. More than 80% of our surviving patients have been decannulated. We have used our data on outcomes to counsel families when deciding upon tracheostomy, as we feel that tracheostomy is a safe and appropriate procedure that can facilitate weaning from mechanical ventilation and transfer out of the ICU.
